# Bacterial co-infections in mucormycosis in severely ill populations: an overlooked and complex challenge

**DOI:** 10.1099/acmi.0.000850.v4

**Published:** 2024-11-12

**Authors:** D. E. Corzo Leon, V. H. Ahumada-Topete, L. Ostrosky-Zeichner

**Affiliations:** 1 Medical Research Council Centre for Medical Mycology, University of Exeter, Exeter, UK; 2 Departamento de Infectología, Instituto Nacional de Enfermedades Respiratorias, Mexico City, Mexico; 3 Division of Infectious Diseases, McGovern Medical School, The University of Texas Health Science Center at Houston, Houston, USA

**Keywords:** bacterial, co-infections, mucormycosis

## Abstract

Mucormycosis is found in co-infection with bacteria in >50% of the cases. Most of these cases were reported among people with haematological diseases. The two most frequent bacteria found were *Pseudomonas aeruginosa* and *Klebsiella pneumoniae*. Almost 40% of the identified bacteria were reported as multidrug resistant.

## Data Summary

Supplementary material contains all references reviewed and included in this report. All analysed data can be found in these references, which are publicly available in PubMed.

## Introduction

Mucormycosis is a devastating invasive fungal infection causing fatal outcomes in >50% of the affected populations [1]. Three main groups of susceptible populations have been recognized as being at the highest risk of mucormycosis: (1) those living with uncontrolled diabetes [1,2]; (2) those deeply immunocompromised by chemotherapy and other immunosuppressants such as corticosteroids, biologics and Chimeric Antigel Receptor T-cell (CART cell) therapy [1,3] and (3) those after suffering penetrating trauma and burns [1,4]. Lately, severely ill individuals with COVID-19 have also been recognized as an emerging group at high risk for this fungal infection [5]. In India alone, this infection caused >40 000 infections, but the association was documented worldwide [6]. Mucormycosis affects mainly rhino-sinus-cerebral areas, lungs and skin, but any site in the body can be affected [1]. Despite the recent awareness of viral co-infections occurring along with invasive mycoses [influenza-associated aspergillosis (IAPA), COVID-19-associated aspergillosis (CAPA) and COVID-19-associated mucormycosis (CAM)] [6,8], bacterial co-infections and their impact on fungal infections and specifically on mucormycosis pathogenesis have been overlooked. A related phenomenon called bacterial endosymbiosis is well described as occurring in Mucorales and plays a major role in fungi-causing diseases in plants [9]. This endosymbiosis is a harmonic interdependent relationship where both sides get biological and evolutionary advantages from the association [10]. This means that not all bacterial species have the capacity to become endosymbionts for these fungi, although many of them can become transitory endofungal bacteria during their interplay [10]. In humans, this association has only been described in less than five cases, and its real frequency is unknown [11,13].

This report aims to contribute to understanding bacterial co-infections in mucormycosis by describing their frequency and clinical characteristics by reviewing previously reported literature.

## Methods

A literature search strategy was carried out in MEDLINE database through PubMed. The following terms were used: ‘zygomycosis’ or ‘mucormycosis’ and ‘co-infections’ or ‘mixed infections’ or ‘bacterial co-infections’. A total of 235 documents were found, from which only those published between 1998 and 2023 were selected. A total of 221 documents were then included, considering the following general co-infection definition. A case of bacterial co-infection in mucormycosis was defined as evidence of bacterial presence in the same anatomic niche as Mucorales within ±14 days of mucormycosis diagnosis. In addition, series/clinical studies reporting co-infection rates were included if they reported co-infection rates in their results using the same case definition. Manuscripts were excluded if no bacterial co-infection was identified, if the type of report was a review or if the text was not fully available online. With the collected information from case series and clinical studies, the frequency of bacterial co-infection occurring in mucormycosis series/clinical studies was estimated and presented as a mean and 95% confidence interval (CI). Clinical characteristics were retrieved where available and presented as frequency (%), median and interquartile range (IQR) as appropriate.

## Results

**Fig. 1.**
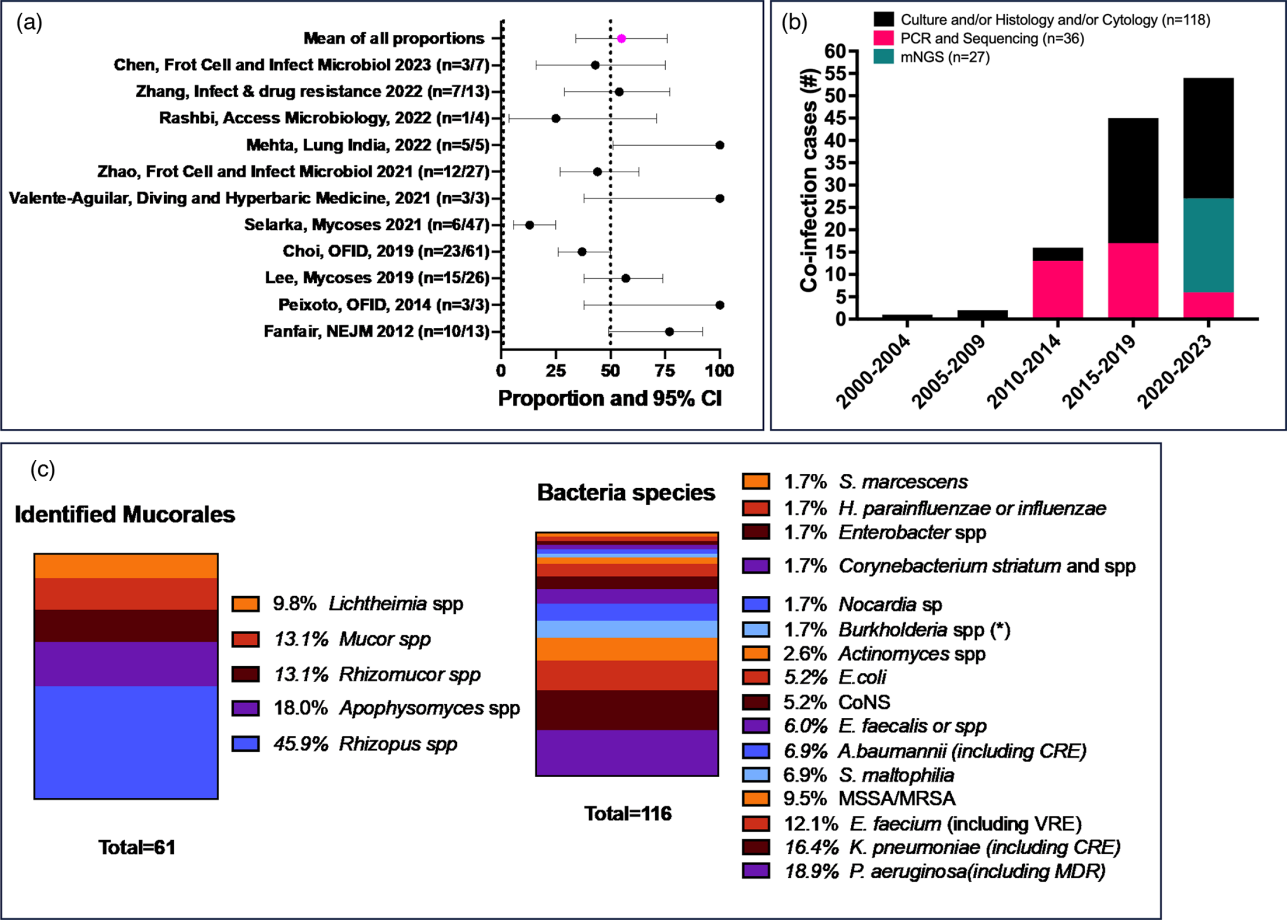
Clinical characteristics of bacterial co-infections in mucormycosis. Evidence found in a literature review spanning 25 years (1998–2023). (a) Summarizing the proportion of bacterial co-infection found in case series or clinical studies reporting mucormycosis cases. (b) Number of cases found in the last 25 years, classified by 5 years of period (no reports found in 1998–1999). Diagnostic tools used in each period (black: histology, cytology, and culture; magenta: PCR and sequencing and green/cyan: mNGS). (c) Fungal and bacterial groups/species identified among the cases of co-infections. *In this category, all *Burkholderia*-like species were included. CoNS, coagulase-negative *Staphylococcus*; VRE, vancomycin-resistant *Enterococcus*; MSSA/MRSA, methicillin-susceptible/methicillin-resistant *Staphylococcus aureus*.

Forty-one manuscripts were included in this review (supplementary table is available with the online version of this article). A total of 118 cases of mucormycosis in co-infection with bacteria were identified among 30 single case reports and 11 case series/clinical studies. The 11 case series/clinical studies were used to estimate the rate of co-infection in mucormycosis. Among these 11 studies, 9 of them reported that bacterial co-infection was not the main aim, but they were reported as one other clinical characteristic of a mucormycosis cohort. Bacterial co-infections in mucormycosis were estimated to occur in 55% (95% CI=34–76%) of the cases (Fig. 1a). Most cases (99/118, 84%) were published between 2015 and 2023. The diagnosis was made using culture and/or histopathology/cytology methods in all cases. Between 2015 and 2023, almost half of the cases were also identified by using PCR/sequencing and/or metagenomics next-generation sequencing (mNGS) approaches (Fig. 1b). In the most recent period (2020–2023), half of the cases were identified using mNGS, either used as a single identifying method or as an additional tool to classical mycological diagnostics (histology, cytology and culture) (Fig. 1b). In clinical studies where only the number of co-infections was provided, it was not possible to obtain further clinical information. Information on clinical syndromes was obtained for 80 cases where bacterial co-infections were reported mainly occurring in pulmonary mucormycosis (*n*=42, 52.5%), followed by rhino-sino-orbital-cerebral (*n*=18, 25.7%) and cutaneous (*n*=16, 8.6%) (Table 1). Most of the bacterial co-infections in mucormycosis were reported among populations with haematological diseases and transplant recipients (45/83, 54%), followed by individuals with diabetes either type 1 or 2 (17/83, 20.5%) and after trauma (15/83, 18%) (Table 1).

**Table 1.** Characteristics of bacterial co-infections in mucormycosis

Characteristics	Frequency *N* (%)
Age (*n*=41, median, IQR)	59, 46–67
Sex (*n*=41)	
‍ Male	27 (57)
Country of report (*n*=118)	
‍ Korea	38
‍ China	27
‍ India	17
‍ USA	16
‍ Portugal	3
‍ Italy	2
‍ Romania	2
‍ Sri Lanka	2
‍ Thailand	2
‍ Others*	9
Clinical presentation (*n*=80)	
‍ Pulmonary	42 (52.5)
‍ Rhino-sino-orbital-cerebral	18 (22.5)
‍ Skin/soft tissue	16 (20.0)
‍ Others†	4 (5.0)
Population at risk (*n*=83)	
‍ Haematological diseases	34 (41)
‍ Diabetes (type 1 or 2)	17 (20.5)
‍ After trauma	15 (18.1)
‍ Transplant recipients	11 (13.3)
‍ After COVID-19‡	9 (11)
Survived (*n*=41)	19 (46)

*Bangladesh, Belgium, Brazil, France, Germany, Greece, Honduras, Iran and Poland (one case each).

†Gastrointestinal (*n*=2) and disseminated (*n*=2).

‡All cases post COVID-19 were in individuals with diabetes.

The most frequently identified fungal species belonged to the genus *Rhizopus* (28/61, 46%), followed by *Apophysomyces* (11/61, 18%), *Rhizomucor* and *Mucor* (both, 8/61, 13.1%) (Fig. 1c). Almost half of the mucormycosis cases were only identified by histological methods, meaning that it is not possible to know neither species nor genera causing the fungal infection (57/118, 48%). Meanwhile, more than 25 bacterial species were identified along with Mucorales. The two bacterial species most frequently isolated were *Pseudomonas aeruginosa* (22/116, 19%) and *Klebsiella pneumoniae* (19/116, 16%) (Fig. 1c). In the *K. pneumoniae* co-infection cases where the outcome was also reported, 7/10 (70%) cases had a fatal outcome compared to 3/11 (27%) of * P. aeruginosa* co-infection. For this review, we considered multidrug resistant (MDR) as resistance to three or more antimicrobial classes [14]. Thus, almost 40% of the identified bacteria were reported as MDR. Endosymbiosis occurs in Mucorales, mostly by *Burkholderia*-like bacteria. These bacterial endosymbionts inhabit environmental fungi and have the capacity of regulating sporulation and producing disease-causing toxins [9,10]. In this review, only two cases associated with *Burkholderia*-like bacteria were found.

Data about demographics and clinical characteristics were identified for 41 cases. The median age among the affected persons was 59 years (IQR 45–67); in general, only one bacterial species was reported along Mucorales (median 1, IQR 1–2), but in three cases, up to five different bacterial species were also found in co-infection with Mucorales in the same event. Empiric antibiotics were used in 58% (24/41) of the cases before the identification or isolation of the bacteria in co-infection. Up to five different antibiotics were administrated before bacteria were identified (mean 2, IQR 1–3, maximum 5). The most frequent type of antibiotic used were beta-lactams (17/53, 32%), mainly piperacillin/tazobactam and cephalosporins, followed by carbapenems (12/53, 22.6%). The overall mortality rate for these co-infections was 46% (*n*=19/41).

## Discussion

The COVID-19 pandemic has brought co-infection research into the spotlight. Numerous research groups are currently focusing on identifying the role of respiratory viral infections on the immune response and clinical outcomes in the presence of fungal pathogens (CAM/CAPA/IAPA). This is undoubtedly a field that needs to be explored to develop future preventive and therapeutic strategies. Despite recognizing the importance of viral–fungal co-infections and cross-kingdom associations, co-infections involving bacteria interacting with fungi, specifically Mucorales, are overlooked, keeping the scale on how these co-infections modify clinical outcomes in mucormycosis as an unexplored field. The current review suggests bacterial co-infections occur in more than half of the mucormycosis cases worldwide. *P. aeruginosa* and *K. pneumoniae* were the two most frequently associated co-infecting bacteria, both being either MDR and/or carbapenem-resistant enterobacterales (CRE). These bacterial species are listed as of high and critical priority (respectively) by the WHO for their ability to transfer antimicrobial mobile and non-mobile elements, the severity and high burden of the infections they cause worldwide in healthcare settings, particularly in Low and Middle Income Countries [15]. In the context of mucormycosis and other fungal infections, it is unknown how cross-kingdom associations contribute to the spreading of antimicrobial resistance and/or the severity of both types of infections, bacterial and fungal. In this review, most cases where *K. pneumoniae* was in co-infection with Mucorales had a fatal outcome. This is a small sample, and further study should be done to understand the impact of the bacterial co-infection on clinical outcomes. In a recent retrospective study by Egge *et al*. [16], they reported similar findings. In their institute, co-infections with Gram-negative bacteria were found to be sharing the same niche in 27% of mucormycosis cases where all affected individuals had leukaemia. The most frequently identified bacteria were *P. aeruginosa*, *Stenotrophomonas maltophilia* and *Enterobacterales* [16]. In immunocompromised populations, these same bacteria are also the most important cause of nosocomial-acquired infections [17]. Considering this feature, most bacterial co-infections in mucormycosis are associated with hospital stay. It is unknown if Mucorales would also be acquired in-hospital or if they were prior lung colonizers and reactivated during the hospital stay. The observations reported here trigger the hypothesis that in hospital settings, both infections should be actively investigated, especially among deeply immunocompromised individuals with the highest probability of developing dual infections.

Research aimed at understanding the complex mechanisms and dynamic relationship between bacteria and Mucorales during mucormycosis, and their impact on pathogenesis and clinical outcomes is required. It is also important to work on the development of new diagnostic and therapeutic strategies considering these co-infections to help in improving outcomes.

The development of new strategies to identify these bacterial–Mucorales infections has different limitations. First, mucormycosis is considered an unusual infection. This misconception leads to disregarding Mucorales as a main cause of disease in certain clinical presentations, such as pulmonary infections. For example, it is known half of pulmonary mucormycosis will be diagnosed only post-mortem [18], which reflects the lack of awareness of the infection and the lack of a pre-mortem biopsy and specific non-invasive diagnostic biomarkers. Another limitation is the low sensitivity of diagnostic tools available to identify mucormycosis: culture, cytology and histopathology [1,19]. New Mucorales-specific diagnostic approaches and highly sensitive tools have been recently developed. These approaches include PCR in tissue [20], bronchoalveolar lavage [21] and serum [22] and mNGS, as well as the recent development of specific antibodies to be used in the point-of-care [23]. The major drawback of these new diagnostic techniques is the global lack of access to them, and some of them have only been used in research contexts.

The identification of the bacterial infection occurs before, at the same time or after mucormycosis is diagnosed. If the identification of a lung bacterial infection occurs before, mucormycosis could be easily missed considering the limitations and the lack of sensitivity and specificity of current diagnostic tools. In addition, the diagnosis of a bacterial infection would be enough to explain pulmonary disease in the first instance, so any further investigation to identify a fungal pathogen will stop. If mucormycosis is also in the same niche, pulmonary disease will not respond or partly respond to antibiotics. The disease would then progress and typically result in a new diagnostic approach that would detect the mucormycosis. However, this means specific treatment for mucormycosis is already late, which increases the risk of fatal outcomes. In the scenario where mucormycosis is diagnosed first, little is known about how frequent underdiagnosed bacterial infections are.

In addition, in these clinical scenarios, the overuse of empirical antibiotics and sometimes antifungals with no specific anti-Mucorales activity is frequent. This will contribute to the emerging and spreading of MDR pathogens.

## Conclusions

The evidence reported here suggests bacterial co-infection in mucormycosis is a very frequent but unrecognized phenomenon. The impact of this interaction on the pathogenesis of mucormycosis is unknown. Our current work aims to define the impact of Mucorales–bacterial interactions on mucormycosis pathogenesis and their role in antimicrobial resistance using cutting-edge molecular approaches. The prompt recognition of this co-infection requires high awareness from clinical teams. Better outcomes could be achieved if new diagnostic strategies are designed to identify these infections. To contribute to these strategies, we are expanding our clinical network to evaluate these interactions prospectively and provide evidence of the use of new biomarkers.

## supplementary material

10.1099/acmi.0.000850.v4 Uncited Supplementary Material 1.

## Abbreviations

CAM: COVID-19-associated mucormycosis
CAPA: COVID-19-associated aspergillosis
CI: confidence interval
CRE: carbapenem-resistant enterobacterales
IAPA: influenza-associated aspergillosis
IQR: interquartile range
MDR: multidrug resistant
mNGS: next-generation sequencing
VRE: vancomycin-resistant Enterococcus

## References

[1] Cornely OA, Alastruey-Izquierdo A, Arenz D, Chen SCA, Dannaoui E (2019). Global guideline for the diagnosis and management of mucormycosis: an initiative of the European Confederation of Medical Mycology in cooperation with the Mycoses Study Group Education and Research Consortium. Lancet Infect Dis.

[2] Corzo-León DE, Chora-Hernández LD, Rodríguez-Zulueta AP, Walsh TJ (2018). Diabetes mellitus as the major risk factor for mucormycosis in Mexico: epidemiology, diagnosis, and outcomes of reported cases. Med Mycol.

[3] Cheok KPL, Farrow A, Springell D, O’Reilly M, Morley S (2024). Mucormycosis after CD19 chimeric antigen receptor T-cell therapy: results of a US Food and Drug Administration adverse events reporting system analysis and a review of the literature. Lancet Infect Dis.

[4] Dang J, Goel P, Choi KJ, Massenzio E, Landau MJ (2023). Mucormycosis following burn injuries: a systematic review. Burns.

[5] Muthu V, Agarwal R, Rudramurthy SM, Thangaraju D, Shevkani MR (2024). Risk factors, mortality, and predictors of survival in COVID-19-associated pulmonary mucormycosis: a multicentre retrospective study from India. Clin Microbiol Infect.

[6] Hoenigl M, Seidel D, Carvalho A, Rudramurthy SM, Arastehfar A (2022). The emergence of COVID-19 associated mucormycosis: a review of cases from 18 countries. *Lancet Microbe*.

[7] van de Veerdonk FL, Kolwijck E, Lestrade PPA, Hodiamont CJ, Rijnders BJA (2017). Influenza-associated aspergillosis in critically ill patients. Am J Respir Crit Care Med.

[8] Alanio A, Dellière S, Fodil S, Bretagne S, Mégarbane B (2020). High prevalence of putative invasive pulmonary aspergillosis in critically ill COVID-19 patients. SSRN J.

[9] Partida-Martinez LP, Groth I, Schmitt I, Richter W, Roth M (2007). *Burkholderia rhizoxinica* sp. nov. and *Burkholderia endofungorum* sp. nov., bacterial endosymbionts of the plant-pathogenic fungus *Rhizopus microsporus*. Int J Syst Evol Microbiol.

[10] Araldi-Brondolo SJ, Spraker J, Shaffer JP, Woytenko EH, Baltrus DA (2017). Bacterial endosymbionts: master modulators of fungal phenotypes. Microbiol Spectr.

[11] Tansarli GS, Eschbacher J, Schroeder LK, SenGupta D, Lieberman JA (2023). *Mycetohabitans rhizoxinica* in patients with rhinocerebral mucormycosis due to *Rhizopus microsporus*. Mycopathologia.

[12] Yang S, Anikst V, Adamson PC (2022). Endofungal *Mycetohabitans rhizoxinica* bacteremia associated with *Rhizopus microsporus* respiratory tract infection. *Emerg Infect Dis*.

[13] Itabangi H, Sephton-Clark PCS, Tamayo DP, Zhou X, Starling GP (2022). A bacterial endosymbiont of the fungus *Rhizopus microsporus* drives phagocyte evasion and opportunistic virulence. Curr Biol.

[14] Magiorakos A-P, Srinivasan A, Carey RB, Carmeli Y, Falagas ME (2012). Multidrug-resistant, extensively drug-resistant and pandrug-resistant bacteria: an international expert proposal for interim standard definitions for acquired resistance. Clin Microbiol Infect.

[15] World Health Organization (2024). WHO bacterial priority pathogens list, 2024: bacterial pathogens of public health importance to guide research, development and strategies to prevent and control antimicrobial resistance.

[16] Egge SL, Wurster S, Cho S-Y, Jiang Y, Axell-House DB (2024). co-occurrence of gram-negative rods in patients with hematologic malignancy and sinopulmonary mucormycosis. J Fungi.

[17] Kreitmann L, Helms J, Martin-Loeches I, Salluh J, Poulakou G (2024). ICU-acquired infections in immunocompromised patients. Intensive Care Med.

[18] Chakrabarti A, Das A, Mandal J, Shivaprakash MR, George VK (2006). The rising trend of invasive zygomycosis in patients with uncontrolled diabetes mellitus. Med Mycol.

[19] Hasan S, Gupta P, Shukla D, Banerjee G (2023). A comparison between potassium hydroxide (KOH) microscopy and culture for the detection of post-COVID-19 rhino-orbital-cerebral mucormycosis. Cureus.

[20] Hammond SP, Bialek R, Milner DA, Petschnigg EM, Baden LR (2011). Molecular methods to improve diagnosis and identification of mucormycosis. J Clin Microbiol.

[21] Scherer E, Iriart X, Bellanger AP, Dupont D, Guitard J (2018). Quantitative PCR (qPCR) detection of mucorales DNA in bronchoalveolar lavage fluid to diagnose pulmonary mucormycosis. J Clin Microbiol.

[22] Millon L, Herbrecht R, Grenouillet F, Morio F, Alanio A (2016). Early diagnosis and monitoring of mucormycosis by detection of circulating DNA in serum: retrospective analysis of 44 cases collected through the French Surveillance Network of Invasive Fungal Infections (RESSIF). Clin Microbiol Infect.

[23] Davies GE, Thornton CR (2022). Development of a monoclonal antibody and a serodiagnostic lateral-flow device specific to *Rhizopus arrhizus* (Syn. *R. oryzae*), the principal global agent of mucormycosis in humans. J Fungi.

